# Volatile Profiling and Variety Discrimination of Leather Using GC-IMS Coupled with Chemometric Analysis

**DOI:** 10.3390/s26020382

**Published:** 2026-01-07

**Authors:** Lingxia Wang, Siying Li, Xuejun Zhou, Yang Lu, Xiaoqing Wang, Zhenbo Wei

**Affiliations:** 1 Zhejiang Institute of Quality Science, 398 Jianshesan Road, Hangzhou 311202, China; neidahappygirl@163.com (L.W.); zhouxuejun119@163.com (X.Z.); zjttjly@outlook.com (Y.L.); silverygirl@163.com (X.W.); 2 Department of Biosystems Engineering, Zhejiang University, 866 Yuhangtang Road, Hangzhou 310058, China; lisiying@zju.edu.cn

**Keywords:** Gas Chromatography–Ion Mobility Spectrometry, leather authentication, volatile organic compounds, multivariate statistical analysis, species discrimination

## Abstract

Volatile fingerprint analysis using Gas Chromatography–Ion Mobility Spectrometry (GC-IMS) was applied to differentiate cowhide (22 samples), sheepskin (6 samples), and pigskin (6 samples). A total of 126 signal peaks were detected from the whole GC-IMS dataset, with 96 volatile compounds identified. Principal Component Analysis (PCA) revealed distinct clustering: cowhide exhibited unique volatile profiles, separating itself clearly from sheepskin and pigskin, which showed significant similarity. This was confirmed by Hierarchical clustering, K-means clustering (optimal k = 2), and Partial Least Squares Discriminant Analysis (PLS-DA) (R^2^ = 0.9836, Q^2^ = 0.9040). Cowhide was characterized by exclusive compounds (2-Hexanone, alpha-Thujene, Butyl acetate, 3-Methyl-2-butanol, 2-Heptanone, Hexyl methyl ether-monomer, Diethyl disulfide). Sheepskin and pigskin shared exclusive compounds (2-Methyl propanol, Isobutyl acetate, 2-Pentyl acetate, 3-Penten-2-one, 2,5-Dimethylfuran). Orthogonal PLS-DA (OPLS-DA) further differentiated sheepskin (Ethyl isobutanoate-dimer, Pentyl acetate-dimer, 3-Methyl-2-butanol, 2-Pentanone, 2-Methylbutanol-dimer, 3-Methyl-1-butanol, 2,5-Dimethylfuran, Propan-2-ol, Ethanol-dimer, and alpha-Thujene) and pigskin (Butan-2-one, Pentanal-dimer, 1-Pentanal-monomer, Ethyl vinyl ether, Z-2-Heptene, and Butyronitrile), identifying alpha-Thujene, 3-Methyl-2-butanol, and 2,5-Dimethylfuran as universal discriminatory markers. GC-IMS coupled with chemometric analysis provides a robust approach for leather authentication.

## 1. Introduction

Leather is widely utilized in daily life. As a polymeric composite material derived from animal hides, leather products exhibit favorable hygiene properties, dyeability, and robust mechanical performance, demonstrating significant potential for use in apparel, footwear, bags, and household goods [1,2]. Natural leathers such as cowhide, sheepskin, and pigskin exhibit substantial performance variations, making them suitable for different manufacturing applications [3]. Their market values also differ considerably, posing challenges for consumer identification [4,5]. Characterizing the composition and concentration of volatile substances in cowhide, sheepskin, and pigskin provides critical safeguards for consumers selecting safe and compliant leather products. This not only protects consumer rights but also assists manufacturers in optimizing production processes. These insights contribute to improved product quality, the reduction of undesirable odors, enhanced customer satisfaction, and strengthened market competitiveness. Furthermore, this research provides data supporting the establishment of relevant industry standards and specifications, enabling effective differentiation of leather product raw materials and components to promote standardized development within the leather industry [6]. Traditional identification methods such as sensory evaluation and infrared spectroscopy have been largely phased out in the market due to their ineffectiveness in distinguishing leather types [4,7]. Meanwhile, optical microscopy identification is cost-prohibitive, technically complex, and challenging for non-specialists to implement accurately [5,8].

Volatile organic compound (VOC) emissions and odor is established as the important indicators for different types of leathers [9,10], because the complex sources of VOCs and odor are produced by its animal-sourced inherent characteristics as a natural product. The flavor analysis techniques like Gas Chromatography–Mass Spectrometry (GC-MS) was applied for the animal species of samples studies, and five different types of Leather (goatskin, sheepskin, horsehide, bovine, and pigskin) were clear classified [11,12]. However, GC-MS provide precise compound identification but require complex sample preparation and are time-consuming [13,14,15]. Although electronic nose detection offers rapid analysis, it exhibits limitations in differentiating samples with similar aromatic profiles [14]. Gas Chromatography–Ion Mobility Spectrometry (GC-IMS), an emerging analytical technique, combines the high separation capability of gas chromatography with the high sensitivity of ion mobility spectrometry [13,14,16]. Requiring minimal sample preparation, featuring straightforward operation, and delivering rapid detection, this technology demonstrates unique advantages in volatile compound analysis and shows significant promise for food and agricultural product authentication, particularly for distinguishing volatile profiles across different leather types [7,13].

Therefore, this study employs GC-IMS technology to analyze the volatile fingerprints of cowhide, sheepskin, and pigskin samples. Through chemometric approaches, we aim to identify marked volatile components for discriminating these three leather types and establish a methodological model for their identification, providing a scientific foundation for classification and authentication within the leather market.

## 2. Materials and Methods

### 2.1. Leather Collection and Sample Preparation

**Figure 1:**
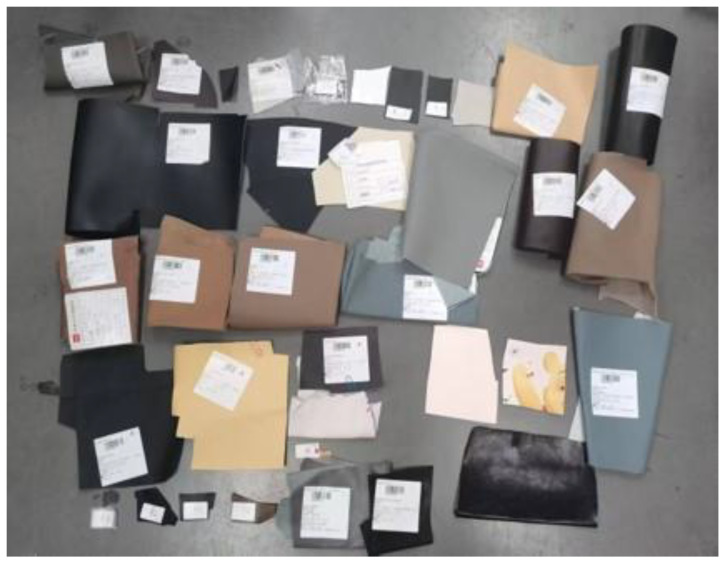
The leather samples.

Twenty-two types of cowhide samples (C6911, C4993, C4996, C5336, C0681, C1867, C5206, C1868, C1866, C1865, C7999, C5207, C4991, C1869, C5337, C5338, C5335, CBN, CYN, CPP, CWN, C0146), six types of sheepskin samples (S5609, S5607, SY1, SY2, SY3, SY4) and six types of pigskin samples (P5333, P5334, PZP, P5330, P1859, PBPG) were provided by the Zhejiang Institute of Quality Science (Hangzhou, China) transported to the laboratory and stored at room temperature for testing (all finished leather, Figure 1).

### 2.2. Volatile Profiles Analysis of Leather

Analysis was performed using a Flavourspec^®^ GC-IMS system (G.A.S., Dortmund, Germany) equipped with an autosampler (CTC Analytics AG, Zwingen, Switzerland) and an Rtx-WAX capillary column (30 m × 0.32 mm × 0.25 μm, RT-12424; Restek, Beijing, China). A 1 g sample of leather, cut into 5 cm × 5 cm pieces was placed in a 20 mL headspace vial (Agilent Technologies, Santa Clara, CA, USA). The headspace vial was incubated at 60 °C with 500 r/min agitation for 30 min. Subsequently, 300 μL of headspace gas was injected into the inlet (80 °C) using a gastight syringe maintained at 85 °C. The column was maintained at 40 °C. Ultra-pure nitrogen (99.99%) carrier gas flow was programmed as follows: initial flow 2 mL/min (hold 2 min), ramped to 10 mL/min over 10 min, ramped to 100 mL/min over 20 min, ramped to 150 mL/min over 30 min. Ionization utilized a β-radiation source (^3^H, 300 MBq) in positive ion mode. The drift tube operated at 45 °C with a 9.8 cm length and 500 V/cm electric field strength, using nitrogen drift gas at 150 mL/min. Retention indices were calibrated using n-ketone standards (C_4_–C_9_; Sinopharm, Beijing, China). Three parallels were set for each sample. Volatile compound identification was achieved by matching against both the NIST database and the GC-IMS Library Search application’s IMS database. The identification of compounds consists of two key steps: first, comparing the retention index of the compound with that of reference standards in the NIST database; second, comparing its drift time with that of standard compounds in the IMS database. If both values show close agreement with those of known standards, the compound can be confidently identified. Data extraction, processing, and generation of topographic plots and comparative gallery plots were performed using LAV software (version 2.2.1; G.A.S., Dortmund, Germany) to obtain signal peaks information.

### 2.3. Statistical Analysis

Principal Component Analysis (PCA) utilized median-based autoscaling normalization (centering on the median and scaling by the standard deviation of each variable [13]) of the raw data implemented in MetaboAnalyst 6.0 (www.metaboanalyst.ca, access date: 20 June 2025). Hierarchical cluster analysis (HCA) is a clustering technique used to group similar objects into clusters based on their pairwise distances or similarities. HCA builds a treelike hierarchical decomposition of the data, where clusters at each level of the hierarchy are formed by merging or splitting existing clusters [17]. K-means minimizes within-cluster squared distances and is simple to implement when using class cardinality, which denotes the number of samples per class [18]. HCA was executed using MetaboAnalyst 6.0 and the h-clust function from the base R package (R 4.4.1). The optimal number of clusters (k) was determined using the within-group sum of squares (WSS) and the silhouette method, implemented via the fviz_nbclust function from the factoextra R package [19,20]. Specific classification results were visualized as heatmaps and PCA score plots. Heatmaps and PCA score plots were generated by MetaboAnalyst 6.0. Partial Least Squares Discriminant Analysis (PLS-DA) and Orthogonal Partial Least Squares Discriminant Analysis (OPLS-DA) were also conducted using MetaboAnalyst 6.0. Other data calculations were performed using Microsoft Excel 2016 (Microsoft Corp., WA, USA).

## 3. Results and Discussion

### 3.1. Volatile Profiles Analysis of Leather with Different Varieties

**Figure 2:**
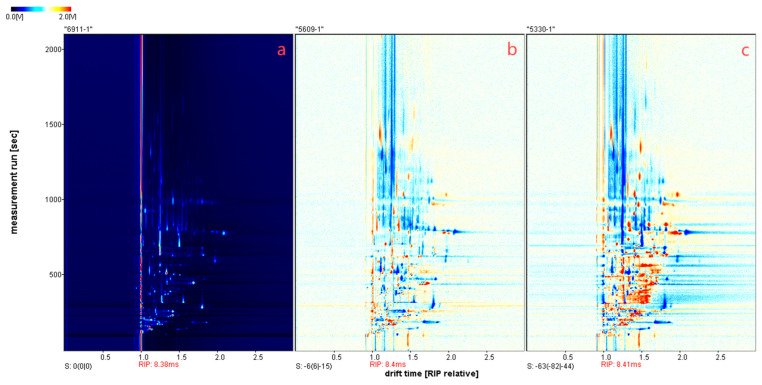
Topographic plot of Cowhide (**a**), Sheepskin (**b**) and Pigskin (**c**) samples detected by GC-IMS (sheepskin and pigskin spectra are processed on the basis of the cowhide reference spectrum by subtracting any background signals shared with it).

**Figure 3:**
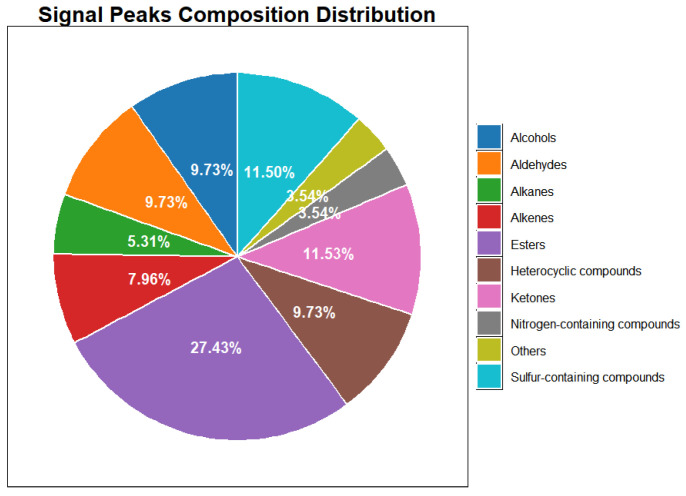
Pie Chart of Volatile Compound Distribution in Leather Samples (Cowhide, Sheepskin, Pigskin) by GC-IMS.

**Figure 4:**
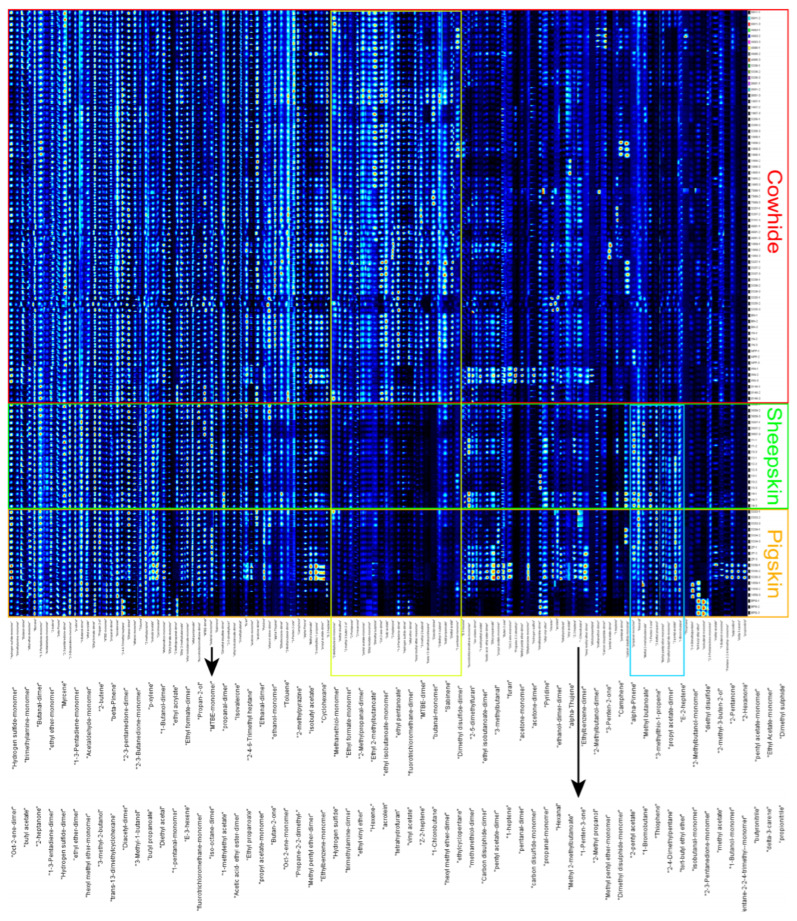
The gallery plot of Cowhide, Sheepskin and Pigskin samples detected by GC-IMS.

A total of 126 signal peaks were extracted from three types of leather samples (Table 1). Approximately 93% of the compounds were located in the front region of the topographic plot (Figure 2), with retention times concentrated between 100–1000 s and retention indices ranging from 400 to 1200. Ninety-six volatile compounds were identified, encompassing esters, alcohols, acids, ketones, aldehydes, alkanes, sulfur-containing compounds, nitrogen-containing compounds, and heterocyclic compounds. Among these, esters constituted the largest proportion at 27.43% (Figure 3). When all signal peaks were arranged in a gallery plot (Figure 4), the volatile fingerprints of sheepskin and pigskin showed significant similarity, while cowhide exhibited distinct differences from both. Three compounds—dimethyl disulphide-monomer, 2-pentyl acetate, and 1-bromobutane—were exclusively present in both sheepskin and pigskin but absent in cowhide. Conversely, hexyl methyl ether-monomer, 3-methyl-2-butanol, and diacetyl-dimer were uniquely detected in cowhide but not observed in either sheepskin and pigskin samples.

### 3.2. Principal Component Analysis of GC-IMS Data

**Figure 5:**
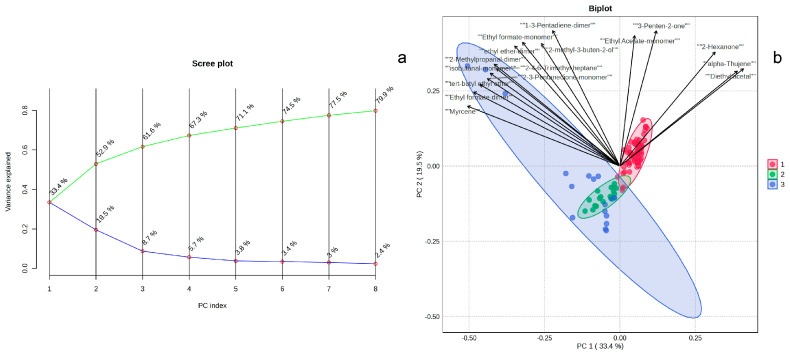
Scree Plot (**a**) and Biplot (**b**) from PCA Based on GC-IMS Results of Cowhide, Sheepskin, and Pigskin (1 refers to Cowhide; 2 refers to Sheepskin; 3 refers to Pigskin).

To further analyze the volatile fingerprint characteristics of leather and identify signature volatile components for discriminating the three leather types, PCA was performed on the GC-IMS data. PCA was performed to visualize the overall volatile profile of leather samples (Twenty-two types of cowhide, six types of sheepskin and six types of pigskin were used for PCA, and three parallels were set for each sample). PCA is an unsupervised dimensionality reduction technique that projects high-dimensional data onto a lower-dimensional space through linear transformation, preserving the directions of maximum variance (principal components) [16]. The first 8 principal components (PCs) cumulatively explained 79.9% of the variance in the odor profiles (Figure 5a). Among these, the first two principal components (PC1 and PC2) individually accounted for 33.4% and 19.5% of the odor profile variance, respectively. In the biplot composed of the score plot and the loading plot (Figure 5b), it can be observed that the positions of the cowhide and sheepskin samples were relatively concentrated, indicating that their volatile fingerprint characteristics were quite similar and the differences among the samples were small. The positions of the pigskin samples were relatively scattered, suggesting that the volatile fingerprint characteristics among the pigskin samples varied significantly. Furthermore, the interaction between chemical agents and the skin matrix varies significantly according to the hide’s initial density, fiber weave tightness, and lipid content during pre-tanning operations, and different volatile profiles can be modulated based on the same raw material. By marking the signal peaks that contributed significantly to PC1, it can be found that except for Ethyl Acetate-monomer, 3-penten-2-one, 2-hexanone, alpha-thujene, and Diethyl acetal which contribute to cowhide, the others all contribute to pigskin and sheepskin.

### 3.3. Construction of Clustering Model and Elimination of Abnormal Samples

**Figure 6:**
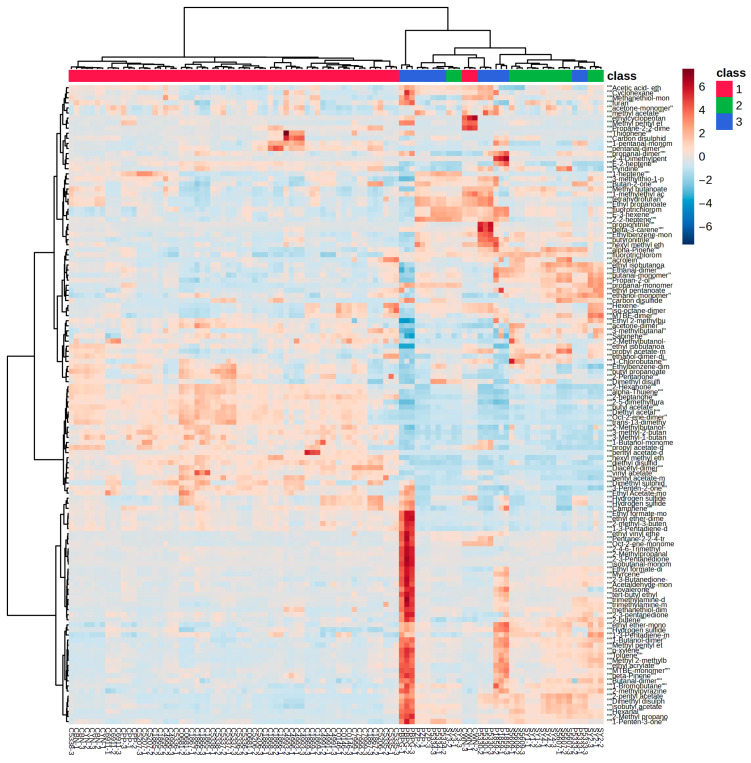
Heatmap of Hierarchical Clustering Analysis of Volatile Compounds in Cowhide, Sheepskin, and Pigskin Detected by GC-IMS (Class 1 refers to Cowhide; Class 2 refers to Sheepskin; Class 3 refers to Pigskin).

Hierarchical clustering and K-means clustering analysis were further employed to identify distinct leather categories. All leather samples were subjected to hierarchical clustering analysis, and the results were presented in the form of a heatmap (Figure 6). It can be observed that the samples were mainly clustered into two groups: one group consisted entirely of cowhide samples, and the other group included one cowhide sample (CWN) and all the sheepskin and pigskin samples. A set of compounds was identified as the key differentiators between cowhide sample CWN and other specimens, including Ethylcyclopentane, Methyl pentyl ether-dimer, Propane-2,2-dimethyl-, Methyl butanoate, 1-methylethyl acetate, Tetrahydrofuran, Ethyl propanoate, Fluorotrichloromethane-dimer, E-3-hexene, and Z-2-heptene. Additionally, the PBPG sample from the pigskin was significantly different from the other leather samples and could be excluded as an outlier in subsequent analyses.

**Figure 7:**
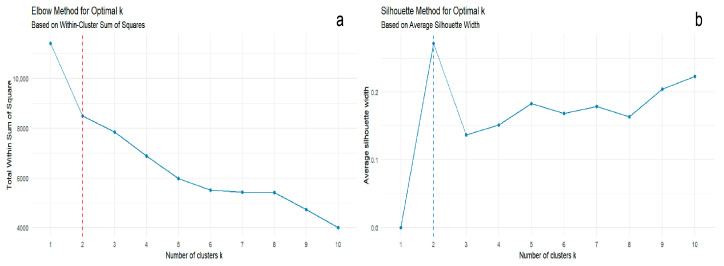
Within Sum of Squares (WSS, (**a**)) and Average Silhouette Width (**b**), used for determining optimal clustering of leather samples (Cowhide, Sheepskin and Pigskin), analyzed by GC-IMS.

**Figure 8:**
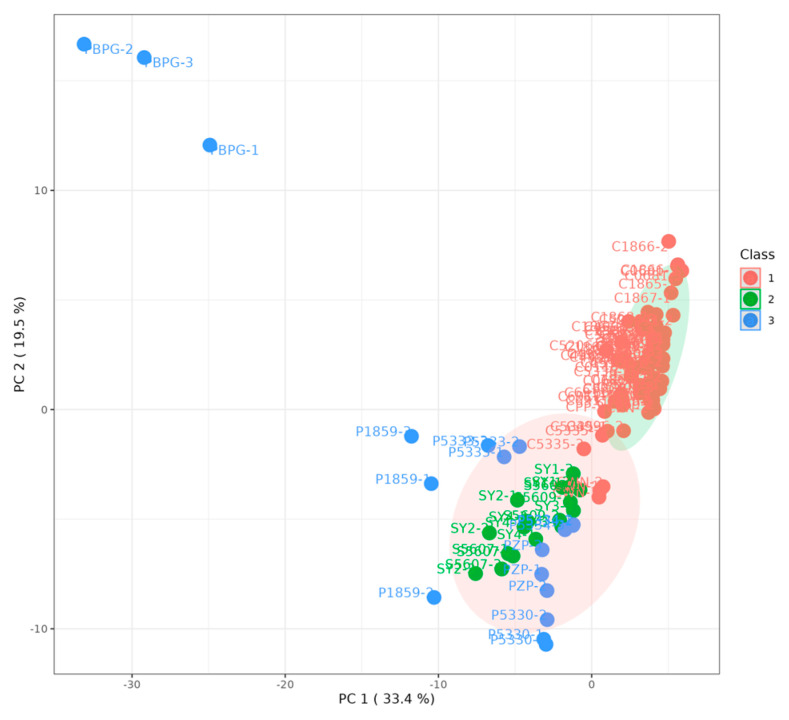
K-means Clustering Results Projected onto the PCA Score Plot of GC-IMS Data from Cowhide, Sheepskin, and Pigskin (Class 1 refers to Cowhide; Class 2 refers to Sheepskin; Class 3 refers to Pigskin).

The optimal number of clusters (k) in K-means clustering analysis was determined using WSS and the silhouette method. WSS is the sum of the squared distances from each sample point to the cluster center [21,22]. Generally, as the number of clusters (cluster centers) increases, the within-cluster sum of squares decreases gradually until it reaches a turning point, after which the rate of decrease slows down. This turning point is usually the optimal number of clusters [19]. The silhouette method assesses the ratio of intra-cluster similarity to inter-cluster similarity [20]. Generally, as the number of clusters (cluster centers) increases, intra-cluster similarity increases gradually and inter-cluster similarity decreases gradually, so the silhouette coefficient increases gradually until it reaches a turning point, after which the rate of increase slows down. This turning point is usually the optimal number of clusters [20]. Therefore, the k value should be the one with a smaller within-cluster sum of squares and a larger average silhouette coefficient. Based on the results of the odor profile score calculation, it was found that the within-cluster sum of squares continued to decrease with the increase of k, and a turning point occurred at k = 2 (Figure 7a), while the average silhouette coefficient reached its maximum at k = 2 (Figure 7b). This indicates that the leather can be clustered into two groups based on the volatile fingerprint characteristics. Therefore, k = 2 was chosen for K-means clustering, and the clustering results were presented in the score plot of principal component analysis (Figure 8). It can also be observed that the cowhide samples were clustered into one group, and the sheepskin and pigskin samples were clustered into another group. Among them, the PBPG sample from the pigskin was located far away from the other leather samples in the figure, confirming that PBPG could be excluded as an outlier.

### 3.4. Construction of Discriminative Model and Screening of Key Signal Peaks

**Figure 9:**
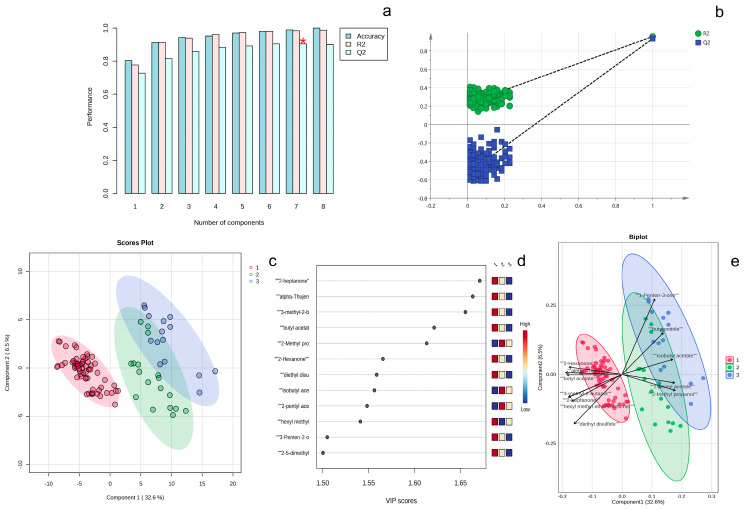
PLS-DA of Cowhide, Sheepskin, and Pigskin based on GC-IMS data: model parameters (**a**), permutation tests (**b**), score plot (**c**), VIP values (**d**) and key signal peaks with VIP > 1.5 projected on score plot (**e**) (1: Cowhide; 2: Sheepskin; 3: Pigskin).

PLS-DA is a supervised classification method that employs a projection-based approach for dimensionality reduction. It is commonly used to build predictive models and visualize the distribution and trends among different sample groups [16]. All samples were subjected to PLS-DA analysis based on leather types after excluding PBPG (Figure 9). The GC-IMS signal peak intensities of all samples were automatically scaled for analysis. Through five-fold internal cross-validation [13], it was found that the first 7 latent variables made significant contributions to the model, so the model was established using the first 7 latent variables (Figure 9a). The goodness-of-fit value (R^2^) of the model constructed by the first 7 latent variables was 0.9836, and the predictive ability parameter (Q^2^) was 0.9040, which was greater than 0.4, indicating that the model was predictive [23]. The robustness of the PLS-DA model was demonstrated through permutation tests (Figure 9b). The score distribution of the three types of leather samples was analyzed using the first 2 latent variables (accounting for 39.1% of the cumulative variance) (Figure 9c). It was found that the cowhide samples could be distinguished from the other two types of samples, but there was some overlap between the pigskin and sheepskin samples. The projection importance variable (VIP) values of each signal peak were calculated to identify those signal peaks that made significant contributions to the discrimination of the three types of leather. A VIP value greater than 1.5 is usually considered an indicator of extremely important variables [24]. In this model, 12 signal peaks had VIP values greater than 1.5 (Figure 9d), indicating their significant contributions to the discrimination of the three types of leather. The above 12 signal peaks were marked on the score plot (Figure 9e). It was found that 7 signal peaks (2-hexanone, alpha-thujene, Butyl acetate, 3-methyl-2-butanol, 2-Heptanone, Hexyl methyl ether-monomer, Diethyl disulfide) mainly contributed to cowhide leather, while the remaining 5 signal peaks (2-methyl propanol, Isobutyl acetate, 2-pentyl acetate, 3-penten-2-one, 2,5-dimethylfuran) contributed to sheepskin and pigskin samples. The differences in volatile organic compound (VOC) profiles among various leathers can be fundamentally attributed to the combined effects of biological characteristics. Genetic factors determine fundamental differences in skin composition and structure. These include variations in collagen cross-linking patterns, keratin types, lipid composition (particularly in subcutaneous fat deposits), and the distribution and secretory activity of sweat and sebaceous glands. For instance, sheepskin’s characteristic lanolin content generates distinct esters and aldehydes upon degradation, while pigskin’s unique follicle structure and dense sweat glands may produce more sulfur- or nitrogen-containing volatile metabolites.

**Figure 10:**
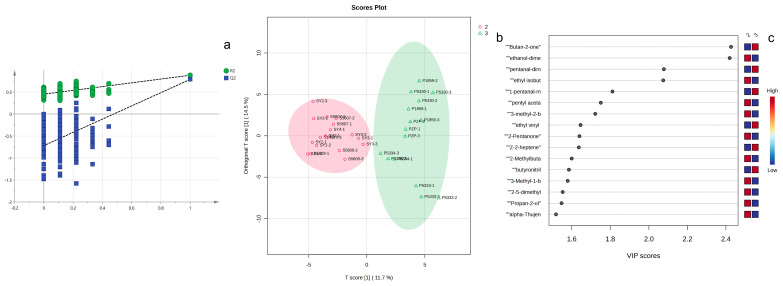
OPLS-DA of Sheepskin and Pigskin by GC-IMS: permutation tests (**a**), score plot (**b**), and VIP values (**c**) (2: Sheepskin; 3: Pigskin).

OPLS-DA is an enhanced multivariate statistical approach specifically designed for binary classification problems and data noise reduction. Its core principle involves decomposing data into predictive variation (associated with class prediction) and orthogonal variation (unrelated to class labels) through Orthogonal Signal Correction (OSC), thereby enhancing model interpretability and robustness [25,26]. To further distinguish the sheepskin and pigskin samples, OPLS-DA was employed for the analysis. An OPLS-DA model was established based on one predictive variable and two orthogonal variables, with the model R^2^ being 0.612 and Q^2^ being 0.525 based on the predictive variable. The robustness of the OPLS-DA model was demonstrated through permutation tests (Figure 10a). According to the score plot (Figure 10b), it can be observed that the sheepskin and pigskin samples can be differentiated based on the predictive variable. Among them, there are 16 signal peaks (VIP > 1.5) that have significant contributions to the predictive variable (Figure 10c). Ethyl isobutanoate-dimer, Pentyl acetate-dimer, 3-methyl-2-butanol, 2-pentanone, 2-methylbutanol-dimer, 3-methyl-1-butanol, 2,5-dimethylfuran, Propan-2-ol, Ethanol-dimer, and alpha-thujene contributed more in the sheepskin samples, while Butan-2-one, Pentanal-dimer, 1-pentanal-monomer, Ethyl vinyl ether, Z-2-heptene, and Butyronitrile contributed more in the pigskin samples. And alpha-thujene, 3-methyl-2-butanol, and 2,5-dimethylfuran not only had significant contributions to the differentiation of the three types of leather but also to the distinction between sheepskin and pigskin samples. Therefore, they can be regarded as key signal peaks for differentiating various types of leather.

## 4. Conclusions

These inherent biochemical constituents form unique “species fingerprints” in VOC profiles. Based on the comprehensive GC-IMS analysis of volatile fingerprints combined with multivariate statistical methods (PCA, HCA, K-means, PLS-DA, OPLS-DA), the three leather types exhibited distinct profiles enabling clear differentiation. Cowhide displayed unique volatile characteristics, significantly differing from both sheepskin and pigskin, as evidenced by its separation in analyses and the exclusive presence of compounds like Hexyl methyl ether-monomer, 3-methyl-2-butanol, and Diacetyl-dimer. Conversely, sheepskin and pigskin showed substantial similarity, forming a distinct cluster separate from cowhide; compounds such as Dimethyl disulphide-monomer, 2-pentyl acetate, and 1-bromobutane were exclusively shared by these two. PLS-DA further confirmed this distinction, identifying key markers like 2-hexanone, alpha-thujene, and Diethyl disulfide as contributing to cowhide, and others like 2-pentyl acetate and 2,5-dimethylfuran as contributing to sheepskin/pigskin. OPLS-DA successfully differentiated the similar sheepskin and pigskin, revealing specific markers for each (e.g., Ethyl isobutanoate-dimer for sheepskin; Butan-2-one, Pentanal-dimer for pigskin). Crucially, alpha-thujene, 3-methyl-2-butanol, and 2,5-dimethylfuran emerged as universal key markers, significantly contributing to the discrimination of all three leather types. The robust statistical models (R^2^ = 0.9836, Q^2^ = 0.9040 ˃ 0.4 for PLS-DA) validated the approach, confirming GC-IMS volatile fingerprinting as a powerful tool for leather type authentication. Nevertheless, this study has several limitations. For instance, the relatively small sample sizes of sheepskin and pigskin may affect the further validation of the model’s stability and predictive performance. Additionally, GC-IMS is restricted to detecting volatile small-molecule compounds, thereby potentially overlooking diagnostic differences that may exist in non-volatile compounds among different leather types. In future work, expanding the sample set to include a greater number and diversity of leather specimens, as well as incorporating complementary techniques such as GC-MS, would help enhance the robustness and broader applicability of the findings.

## Figures and Tables

**Table 1:** Summary of signal peaks identified by GC-IMS.

Count	Compound	CAS	RI ^α^	Rt (s) ^β^	Dt (ms) ^γ^	Comment
1	Propane, 2,2-dimethyl-	463-82-1	379.9	108.116	1.3318	
2	Hydrogen sulfide	7783-06-4	455.4	129.271	1.5488	
3	Hydrogen sulfide	7783-06-4	484.4	138.855	1.143	monomer
4	Hydrogen sulfide	7783-6-4	491	141.179	1.5009	dimer
5	Trimethylamine	75-50-3	544.8	162.041	1.0833	monomer
6	Trimethylamine	75-50-3	545	162.131	1.0521	dimer
7	Fluorotrichloromethane	83589-40-6	578.3	177.02	1.2807	dimer
8	2,4-dimethylpentane	108-08-7	581.3	178.408	1.33	
9	Ethyl ether	60-29-7	586	180.702	1.0579	monomer
10	Fluorotrichloromethane	83589-40-6	594.9	185.07	1.2418	monomer
11	Ethyl ether	60-29-7	595	185.093	1.7039	dimer
12	1,3-pentadiene	504-60-9	597.1	186.151	1.8622	dimer
13	(E)-3-hexene	13269-52-8	613.6	194.646	1.2246	
14	Ethyl vinyl ether	109-92-2	617	196.448	1.2858	
15	1,3-pentadiene	504-60-9	617	196.463	1.1814	monomer
16	Pentane, 2,2,4-trimethyl-	540-84-1	622.6	199.5	1.3717	monomer
17	Methyl tert-butyl ether (MTBE)	1634-04-4	632.3	204.879	1.1084	monomer
18	Acetaldehyde	75-07-0	644.6	211.924	1.0176	monomer
19	Methanethiol	74-93-1	647.7	213.724	1.0416	monomer
20	Methyl tert-butyl ether (MTBE)	1634-04-4	662.3	222.569	1.1165	dimer
21	Hexene-	592-41-6	665.2	224.396	1.3474	
22	Isooctane	540-84-1	673.7	229.822	1.3768	dimer
23	Ethanal	75-07-0	681.8	235.061	1.4181	dimer
24	Dimethyl sulphide	75-18-3	682.2	235.377	1.4999	
25	2-butene	107-01-7	683.1	235.927	1.2078	
26	tert-Butyl ethyl ether	637-92-3	688.4	239.501	1.0649	
27	Carbon disulfide	75-15-0	692.9	242.564	1.096	monomer
28	Methanethiol	74-93-1	697.6	245.836	1.0462	dimer
29	Carbon disulphide	75-15-0	706.4	252.009	1.239	dimer
30	(E)-2-heptene	14686-13-6	717.5	260.128	1.4338	
31	1-heptene	592-76-7	719.5	261.63	1.2951	
32	Propanal	123-38-6	722.5	263.88	1.0749	monomer
33	Propanal	123-38-6	725.1	265.854	1.1928	dimer
34	Cyclohexane	110-82-7	732.2	271.286	1.1105	
35	Methyl pentyl ether	628-80-8	745.2	281.703	1.029	monomer
36	Methyl pentyl ether	628-80-8	769.3	302.063	1.238	dimer
37	ethylcyclopentane	1640-89-7	769.7	302.375	1.6186	
38	Ethyl formate	109-94-4	773.4	305.734	1.812	monomer
39	(Z)-2-heptene	6443-92-1	778.5	310.276	1.1988	
40	Methyl acetate	79-20-9	782.7	314.139	1.5219	
41	Furan	110-00-9	798	328.564	1.473	
42	Isobutanal	78-84-2	798.6	329.101	1.3678	monomer
43	2-methylpropanal	78-84-2	798.7	329.244	1.1604	dimer
44	Acetone	67-64-1	806.4	336.792	1.5628	monomer
45	Butanal	123-72-8	811.3	341.676	1.2846	dimer
46	1-methylethyl acetate	108-21-4	836.9	368.634	1.1769	
47	Oct-2-ene	111-67-1	839	370.919	1.315	dimer
48	Acetic acid, ethyl ester	141-78-6	841.2	373.393	1.3519	dimer
49	Butanal	123-72-8	841.3	373.457	1.0976	monomer
50	1-chlorobutane	109-69-3	842.5	374.798	1.4096	
51	Acetone	67-64-1	842.9	375.236	1.1251	dimer
52	Oct-2-ene	111-67-1	850.9	384.331	1.4867	monomer
53	2,4,6-trimethyl heptane	2613-61-8	855.9	390.118	1.3973	
54	Ethyl formate	109-94-4	856.5	390.875	1.1718	dimer
55	Ethyl acetate	141-78-6	856.8	391.176	1.3159	monomer
56	Acrolein	107-02-8	864.1	399.795	1.0715	
57	3-methylbutanal	590-86-3	872.6	410.114	1.1927	
58	Butan-2-one	78-93-3	877.8	416.684	1.2697	
59	trans-1,3-dimethylcyclohexane	2207-03-6	879.9	419.259	1.4252	
60	Diethyl acetal	105-57-7	881.6	421.385	1.1399	
61	Ethyl propanoate	105-37-3	896.8	441.186	1.6222	
62	tetrahydrofuran	109-99-9	900	445.476	1.6434	
63	Ethyl isobutanoate	97-62-1	901.8	447.994	1.6911	monomer
64	1-pentanal	110-62-3	907.5	455.769	1.4088	monomer
65	Pentanal	110-62-3	913.9	464.679	1.4319	dimer
66	Hexyl methyl ether	4747-07-3	915.5	466.98	1.2256	dimer
67	Vinyl acetate	108-05-4	919	471.871	1.622	
68	Diacetyl	431-03-8	920.7	474.298	1.8371	dimer
69	1-bromobutane	109-65-9	921.9	476.124	1.0954	
70	Hexyl methyl ether	4747-07-3	923	477.676	1.3968	monomer
71	Propan-2-ol	67-63-0	938.5	500.715	1.1769	
72	Propyl acetate	109-60-4	939.1	501.693	1.5123	monomer
73	Ethyl isobutanoate	97-62-1	941.8	505.795	1.4342	dimer
74	2,5-dimethylfuran	625-86-5	946.9	513.048	1.3411	
75	2-pentanone	107-87-9	952.2	522.155	1.1397	
76	Methyl butanoate	623-42-7	953.2	523.81	1.2479	
77	Ethanol	64-17-5	957.2	530.329	1.1107	monomer
78	Ethanol	64-17-5	967.6	547.455	1.144	dimer
79	3-(methylthio)-1-propene	10152-76-8	978.8	566.602	1.5189	
80	Propyl acetate	109-60-4	980.1	568.886	1.591	dimer
81	Ethyl 2-methylbutanoate	7452-79-1	984.3	576.38	1.2498	
82	Thiophene	110-02-1	993.4	592.674	2.0955	
83	2-methyl-3-buten-2-ol	115-18-4	999.2	603.426	1.982	
84	2,3-butanedione	431-03-8	1009.5	622.947	1.1548	monomer
85	1-penten-3-one	1629-58-9	1011.3	626.475	1.456	
86	Isobutyl acetate	110-19-0	1012.2	628.154	1.6298	
87	Ethyl acrylate	1408-8-5	1016.2	635.904	1.4152	
88	Alpha-pinene	80-56-8	1019.3	642.148	1.8121	
89	Methyl 2-methylbutanoate	868-57-5	1028	659.752	1.0758	
90	alpha-thujene	2867-05-2	1036.1	676.501	1.8107	
91	Propionitrile	107-12-0	1039.7	682.903	1.6689	
92	Butyl acetate	123-86-4	1050.7	707.915	1.4015	
93	2,3-pentanedione	600-14-6	1050.9	708.423	1.2415	monomer
94	2-methyl propanol	78-83-1	1051.4	709.576	1.1786	
95	Toluene	108-83-8	1059.9	728.594	1.0302	
96	Butyronitrile	109-74-0	1063.6	737.001	1.3336	
97	2-hexanone	591-78-6	1064.7	739.541	1.5034	
98	beta-pinene	127-91-3	1081.6	779.633	1.4939	
99	Camphene	79-92-5	1082.8	782.662	2.1019	
100	Dimethyl disulphide	624-92-0	1084	785.563	1.9874	monomer
101	2-pentyl acetate	53496-15-4	1084.4	786.42	1.9273	
102	3-penten-2-one	625-33-2	1098.8	822.725	1.533	
103	2,3-pentanedione	600-14-6	1099.9	825.49	1.2307	dimer
104	delta-3-carene	13466-78-9	1103.6	835.155	1.8255	
105	Ethylbenzene	100-41-4	1104.6	837.785	1.5932	monomer
106	Ethyl pentanoate	539-82-2	1108.1	846.941	1.067	
107	Sabinene	3387-41-5	1109.1	849.775	1.3507	
108	3-methyl-2-butanol	598-75-4	1111.8	856.801	1.4296	
109	1-butanol	71-36-3	1121.1	882.027	1.3987	monomer
110	1-butanol	71-36-3	1135.5	922.724	1.165	dimer
111	Butyl propanoate	590-01-2	1136.8	926.422	1.2831	
112	p-Xylene	106-42-3	1138.7	932.193	1.0633	
113	Hexanal	66-25-1	1150.1	966.019	1.5509	
114	Pentyl acetate	628-63-7	1157.3	987.804	1.7517	monomer
115	Isovalerone	108-83-8	1158	989.942	1.8	
116	Myrcene	123-35-3	1159.2	993.738	1.4226	
117	Ethylbenzene	100-41-4	1159.3	994.188	1.655	dimer
118	Dimethyl disulfide	624-92-0	1161.9	1002.07	1.1443	dimer
119	Pyridine	110-86-1	1173.4	1038.763	1.4065	
120	2-methylbutanol	137-32-6	1176.2	1047.912	1.2216	dimer
121	Pentyl acetate	628-63-7	1201.3	1133.621	1.7843	dimer
122	2-heptanone	110-43-0	1204.4	1144.726	1.2709	
123	2-methylbutanol	137-32-6	1219.1	1198.459	1.1739	monomer
124	3-methyl-1-butanol	123-51-3	1220.9	1205.32	1.2351	
125	Diethyl disulfide	110-81-6	1255.2	1341.576	1.13	
126	2-methylpyrazine	109-08-0	1272.8	1417.545	1.1038	

α: The retention index values of compounds on Rtx-WAX capillary column. β: The retention time (s) of compounds on Rtx-WAX capillary column. γ: The drift time (ms) of compounds in the drift tube.

## Data Availability

The raw data supporting the conclusions of this article will be made available by the authors on request.
